# Physico-Chemical Property, Sensory Profile and Consumer Acceptability of Water Buffalo (*Bubalus bubalis* L.) Chocolate Milk Using Alkalized and Natural Cocoa Powder

**DOI:** 10.3390/foods12091797

**Published:** 2023-04-26

**Authors:** Joel G. Juvinal, Hans De Steur, Joachim J. Schouteten, Dimas Rahadian Aji Muhammad, Alma A. De Leon, Koen Dewettinck, Xavier Gellynck

**Affiliations:** 1 Department of Agricultural Economics, Ghent University, Coupure Links 653, 9000 Ghent, Belgium; 2 Sensolab, Faculty of Bioscience Engineering, Ghent University, Coupure Links 653, 9000 Ghent, Belgium; 3 Department of Food Science and Technology, College of Home Science and Industry, Science City of Munoz, Nueva Ecija 3120, Philippines; 4 Department of Food Science and Technology, Universitas Sebelas Maret (UNS), Jl. Ir Sutami 36A Kentingan Jebres, Surakarta 57126, Indonesia; 5 Department of Food Technology, Safety and Health, Food Structure & Function Research Group (FSF), Faculty of Bioscience Engineering, Ghent University, Coupure Links 653, 9000 Ghent, Belgium

**Keywords:** water buffalo, chocolate milk, cocoa powder, sensory analysis, consumer testing, proximate analysis, acceptability, drivers of liking

## Abstract

Due to its nutritional quality and palatability, chocolate milk beverages are gaining popularity globally. Alkalized cocoa powder is mostly used in its production since it provides for more intense color and dispersibility, but it has a negative effect on the phytochemical content of cocoa powder. Studies have suggested that water buffalo milk is superior to other milk due to its higher protein content and superior emulsion properties. As such, this study investigated the physico-chemical characteristics, sensory profile, and consumer acceptability of commercial and prototype water buffalo chocolate milk incorporated with alkalized and natural cocoa powder. Based on four buffalo chocolate milk samples, consumer acceptance was assessed by 120 consumers, which was combined with descriptive sensory data using a trained panel (*n* = 8) to determine drivers of liking. Compositional proximate analysis of alkalized and natural cocoa powders showed a significant difference in pH, moisture content, ash content, and fat content. Descriptive analysis showed that 8 out of 13 attributes (color, visual sandiness, cocoa and vanilla aroma, smoothness, creaminess, vanilla taste, and chocolate aftertaste) were effective discriminators of sensory attributes. Overall, buffalo chocolate milk samples were equally liked, but hedonic ratings of the individual attributes revealed that the samples were statistically different for color, viscosity (mouthfeel), and chocolate flavor. Partial least square regression (PLSR) identified chocolate flavor, viscous appearance, viscous mouthfeel, and bitter aftertaste as positive “drivers of liking”. The darker color provided by alkalized cocoa powder did not increase consumer liking. The purchase intention was equal for all chocolate milk samples, whether alkalized or natural. Both cocoa powders showed comparable performance in the manufacture of buffalo chocolate milk. Using natural cocoa powder may be beneficial to local producers of cocoa powder and cocoa farmers since it is easier to produce, while it can provide a marketing advantage for dairy beverages in the global trend of going back to “organic” and “natural”.

## 1. Introduction

Flavored milk is a delectable and nutrient-rich beverage choice, and its production is one of the most effective ways to increase milk intake in a diet [1,2]. It has been recommended as a beverage option by the American Heart Association and the American Academy of Pediatrics because it may increase the nutrient intake of calcium, vitamin D, and potassium [3,4]. One of the variants of flavored milk that has garnered scientific attention due to its potential nutritional benefits and global popularity is chocolate milk [5,6]. Most chocolate milk products are made from cow’s milk since it accounts for 81% of all kinds of milk produced worldwide [7,8].

Buffalo milk, a dairy product derived from the water buffalo (*Bubalus bubalis* L.), also serves as an essential source of nutrition, and globally it is second only to cow’s milk in terms of popularity and consumption [9,10]. In Asia, where 60% of the world’s population live [11], water buffalo plays a vital part in community development through its contribution as a source of meat, milk, and draft power for farm operations [12]. Almost all (98.03%) of the 203 million buffaloes in the world are in Asia [13]. In the last decade, water buffaloes have been transformed into high-yielding producers of milk and meat by organized crossing and backcrossing with the riverine type [14]. Studies have suggested that buffalo milk may offer several advantages over other milk, including higher protein content, superior emulsion property, and defense against pathogens due to immunomodulatory proteins [15,16,17,18,19]. In terms of food technology, buffalo milk is a promising source of innovative products because it contains high fat and protein, which makes it ideal for processing [9,20,21,22].

Chocolate milk products in the market differ greatly in sensory properties, which is mainly due to the type of milk and the cocoa powder used [23,24]. Cocoa powder contains 10–26% fat, and the properties of cocoa powder (fat content, alkalinity, and color) influence the physical and sensory properties of final products [25]. The consumption of cocoa and its derivatives may have a role in the promotion of health, with evidence pointing to immediate benefits for vascular parameters and cardiovascular function, particularly when flavonoids are present in substantial concentrations [26]. However, the beneficial character of cocoa derivatives is affected by processing resulting in the potential decrease of its health-promoting benefits [27]. A case in point is alkalization, which is also referred to as “Dutching”, which is a supplemental but crucial stage in the manufacturing of cocoa that intensifies its brown color, alters its flavor, and boosts its natural solubility [28]. However, it has several adverse effects in terms of nutrition. High alkali concentrations have a negative effect on phytochemical content [29]. A plethora of research also shows that cocoa powders that are alkalized have significantly lower phenolic compounds, thereby reducing their antioxidant capacity [30,31,32,33]. This is because the increased pH in heavily alkalized cocoa powder causes the oxidation and further degradation of phenolic compounds [34]. Alkalization also adversely affects volatile flavor compounds such as methylxanthines and flavan-3-ols [35]. As such, the use of natural cocoa powder in chocolate milk is an advantage both for nutritional and practical reasons since it resonates with the increased demand for authentic, local, and organic foods with no or fewer synthetic additives [36,37,38,39,40]. Although several studies have analyzed different aspects of cocoa and chocolate-based beverages, this product category is still relatively under-studied [41], whereas the effects on the sensory characteristics and consumer acceptability of buffalo chocolate milk made with natural and alkalized cocoa powder remain to be investigated.

Therefore, this study was conducted to determine the sensory profile and consumer acceptability of chocolate milk drinks from buffalo (*Bubalus bubalis* L.) milk, to determine the effect of the type of cocoa powder on the physico-chemical characteristics, sensory attributes, and acceptability of buffalo chocolate milk, and to identify the drivers of consumer liking based on combined sensory and consumer data.

## 2. Materials and Methods

### 2.1. Characterization of Cocoa Powders

Commercial and prototype samples investigated in this study made use of two types of cocoa powders (for a total of four samples). Natural cocoa powder (Ricoa, Commonwealth Foods, Inc., Manila, Philippines) and alkalized cocoa powder (Valmarce Food Marketing Corporation, Bulacan, Philippines) were used. The cocoa powders were evaluated in terms of color, pH, moisture content, ash content, and crude fat content.

#### 2.1.1. Determination of Color

Munsell Colour System (Munsell color file, Baltimore, MD, USA) was used to determine the color of cocoa powder. Cocoa powder samples were visually compared to a Munsell color file. In order to avoid metameric matches, all eight (8) descriptive sensory panelists were trained to evaluate the color of the cocoa powders. They took the Farnsworth-Munsell 100-hue test to detect if they have color vision deficiency. Afterwards, each panelist was seated in a sensory booth with daylight fluorescent light directly above the samples where the viewing angle of the evaluator was about 45° to the sample. The background color of the viewing area of the booth was matte off-white. Since there was only one set of Munsell color strips, the evaluators conducted this one by one. They first evaluated the hue (5 YR), and then the Munsell color strips of that particular hue were laid out in descending order from top to bottom to match the value (8—light to 2.5—dark). Next, they evaluated from left to right to determine the chroma (vividness from 1 to 8). Their evaluation results were compared with each other, and differences in the evaluation were resolved through consensus.

#### 2.1.2. Determination of pH

The pH of the cocoa powder was determined by weighing 5 g dissolved into 100 mL distilled water (Absolute, Asia Brewery, Cabuyao, Laguna, Philippines) in a 500 mL capacity beaker. The pH meter (Milwaukee Instruments, Inc., Rocky Mount, NC, USA) was calibrated prior to analysis. Three (3) replications were conducted per sample (AOAC, 2012).

#### 2.1.3. Determination of Moisture Content

The moisture content of the cocoa powder was determined by weighing approximately 1.5 g of cocoa powder and placing it in a weighted crucible. The weighed sample was placed into a cabinet oven (Jiangyin Yinghai, Beihai, China) at 101–105 °C and dried for 5 h, and then samples were removed and cooled into the desiccator and then weighed into the analytical balance. The oven drying process was continued for 1 h and 30 min until successive weightings differed by less than 0.1% of the original mass of the cocoa powder. The moisture content was computed using Equation (1):

(1) $$\%\;moisture\;=\frac{\left(initial\;weight\;of\;crucble+filter\;paper)-(final\;weight\;of\;crucible+filter\;paper\right)}{initial\;weight\;of\;sample}\times 100$$

#### 2.1.4. Determination of Ash Content

The local cocoa powder that was used in this study was subjected to an ash content test. Moisture-free cocoa powder was placed into a muffle furnace (Blue M Electric Company, New Columbia, PA, USA) at about 550 °C for 5 h and left until a light gray ash resulted. The sample was cooled in the desiccator and was weighed again. The percent ash was calculated using Equation (2):

(2) $$\%\;ash=\frac{weight\;of\;ash}{weight\;of\;sample}\times 100$$

#### 2.1.5. Determination of Crude Fat Content

Crude fat content determination of the local cocoa powder was carried out by means of a Soxhlet extractor (Thermo Fischer Scientific, Hemel Hempstead, UK). Approximately 1.5 g of moisture-free cocoa powder was placed into the filter paper. It was then transferred to an extraction thimble, plugged lightly with cotton wool, and placed into the extractor. The petroleum was added until it siphoned over, at which point more petroleum was added until the barrel of the extractor was half full and was then placed in a boiling water bath. The heat was adjusted so that the solvent boiled gently, and the system was left to siphon at least 10 times. The extraction was then carried out for 6 h. After extraction, the filter paper containing fat-free samples was weighed and then the percentage of fat was calculated using Equation (3):

(3) $$\%\;fat=\frac{\left(weight\;of\;sample+filter\;paper\right)-weight\;after\;extraction}{weight\;of\;sample}\times 100$$

### 2.2. Rapid Proximate Analysis of Raw Water Buffalo Milk

Analysis of the physico-chemical properties of raw buffalo milk was carried out using an automatic milk analyzer (Foss, Hilleroed, Denmark). About 20–30 mL of raw milk was placed into the vials and was placed on the milk analyzer in duplicate. After 12 min, the results of its compositional properties were obtained.

### 2.3. Preparation of Chocolate Milk Prototype and Selection of Commercial Buffalo Chocolate Milk Samples

For each formulation of chocolate milk, about 3 L (30%) of fresh buffalo milk was measured using a graduated cylinder which was placed in a stainless steel container subjected to medium heat until it reached 63–65 °C. When the buffalo milk reached the desired temperature, about 5.6 L (56.5%) of mineral water (Absolute) was added, followed by an additional 100 g (1%) of cocoa powder, 750 g (7.5%) table sugar (Victoria), 500 g (5%) skimmed milk (NZMP), and 2.5 g (0.025%) stabilizer (Carrageenan). The heat was brought up from 65 °C to 75 °C. After boiling, the hot mixture was strained using cheesecloth to make sure that there were no lumps. The chocolate milk was cooled down to around 20–25 °C in an ice bath before bottling. High-density polyethylene bottles were disinfected prior to chocolate milk production using 15 mL of bleach (Zonrox) per 3.8 L of water. Buffalo chocolate milk was carefully transferred and sealed in clean bottles and were stored in the freezer to prevent spoilage at least 1 d prior to evaluation.

There are currently two commercial brands of buffalo chocolate milk in the Philippine market: PCC (Philippine Carabao Center, Science City of Munoz) and DVF (DVF, Talavera, Nueva Ecija, Philippines). These were selected in this study, and based on personal communication with the manufacturers, the formulation and processing of commercial chocolate milk are similar since it was standardized by the Philippine Carabao Center. Table 1 shows the identity of the samples and the type of cocoa powder that was used in the preparation of the chocolate milk samples.

### 2.4. Physico-Chemical Analysis of Chocolate Milk

#### 2.4.1. Determination of Color and pH

Munsell Color System (Munsell color file, Baltimore, MD, USA) was used to determine the color of chocolate milk following the procedure used for cocoa powder. Twenty to thirty milliliters (20–30 mL) of chocolate milk was measured and placed into a beaker. The end of the pH electrode was dipped into the chocolate milk sample, and the pH was determined and recorded.

#### 2.4.2. Determination of Total Soluble Solids

Twenty to thirty milliliters of chocolate milk were measured and placed into a beaker. The end of the digital refractometer (Atago, Tokyo, Japan) was dipped into the chocolate milk sample, and the total soluble solids (°Brix) was determined and recorded.

#### 2.4.3. Determination of Titratable Acidity

Chocolate milk was tempered at room temperature, and approximately 8.7 mL was measured and placed in an Erlenmeyer flask. A 50 mL of distilled water and 0.5 mL of 1% phenolphthalein solution were added to the solution and was titrated using 0.1 N NaOH until an endpoint of faint pink color was observed. Volume was recorded, and a blank titration was run. Titratable acidity was computed using Equation (4):

(4) $$\%\;Acid=\frac{\left(ml\;base\;titrant\right)\times \left(N\;of\;base\;in\frac{mol}{L}\right)\times Eq.Wt.of\;acid}{\left(sample\;volume\;in\;mL\right)\times 10}$$

#### 2.4.4. Determination of Fat Content

Eighteen grams (18 g) of chocolate milk, 2 mL of ammonium hydroxide, and 3 mL of n-butyl alcohol were placed in a Babcock bottle and were thoroughly mixed. Then, a diluted sulfuric acid (17 mL) was added, and the Babcock bottle was shaken in a rotary motion to enable the charring of protein, carbohydrates, and other components except for fat. Then, samples were centrifuged (Garver Manufacturing Co., Union City, IN, USA) for 7 min. After centrifugation, hot water was added until the fat column was within the graduation. Then, the sample was centrifuged for a final 1 min and warm water was poured into the bottle for 3 min. The sample was tempered to uniformly measure and record the fat column from the graduated scale of the Babcock bottle.

#### 2.4.5. Determination of Protein Content

The protein content of chocolate milk was determined using a Kjeldahl Protein Analyzer (Velp Scientifica, Shanghai, China). The Kjeldahl method consists of three steps: digestion, distillation, and titration. Five milliliters (5 mL) of chocolate milk samples, 12–15 mL of concentrated sulfuric acid, 2 tablets of copper, and 5 mL of 30% hydrogen peroxide were placed into a digestion flask. Then, the digestion flask with the mixture was brought to a rolling boil (about 370–400 °C) using a heating block until fumes could be seen. The flask was cooled, and 250 mL of water was carefully added. After completing the digestion, the digestion flask was connected to a receiving flask by a tube. The solution was then made alkaline by adding 32% NaOH, which converted the ammonium sulfates into ammonia gas and was followed by the addition of 30 mL boric acid solution (4%) and 3 drops of Tashiro’s indicator. Afterwards, the color changed from green to brown when it was subjected to a distillation process for more than 2 min. Finally, the samples were titrated using hydrochloric acid to reach the endpoint by changing their color from brown to purple, indicating that all the acids had been neutralized by the base. After titration, the percent protein was computed using Equations (5) and (6):

(5) $$\%\;nitrogen\left(N\right)=Normality\;of\;HCl\times \frac{corrected\;acid\;volume}{weight\;of\;sample}\times 6.38\times 100$$

(6) % protein=% N×3.38

### 2.5. Sensory Descriptive Analysis of Chocolate Milk

Sensory profiling of chocolate milk was conducted based upon the Generic Descriptive Analysis Method [42] using trained panelists from Central Luzon State University (CLSU), Science City of Munoz, Philippines, with at least two years of sensory evaluation experience. The phases of descriptive analysis were as follows: a qualitative process of lexicon development; and a quantitative set of sensory tests intended to rate the intensity of the sensory terms developed in the lexicon generation phase.

#### 2.5.1. Generation of Descriptors (Lexicon Development)

The selected panelists (*n* = 8) were trained at the CLSU Sensory Laboratory for the sensory profiling of chocolate milk. These panelists had past experience in sensory evaluation and were retrained for chocolate milk evaluation. The panelists were asked by the panel leader to individually characterize each of the four samples of chocolate milk in terms of appearance, aroma, taste, and aftertaste. Sensory attributes/terms that were considered important were noted. After the panelists exhausted all the possible attributes of the samples, similar words were grouped, and the best words that describe each attribute were retained and were placed in an evaluation sheet. Definitions or descriptions of the characteristics were formulated in consensus by the panel.

#### 2.5.2. Referencing of Generated Sensory Descriptors

The panelists received additional training on identifying and rating intensity levels using reference standards. The panel came to an agreement on a scorecard that incorporated all relevant features in the order that they were assessed using an unstructured 15 cm line scale. The training was continued until the panel leader determined that all panelists were able to identify and rate the intensity of each attribute.

#### 2.5.3. Replicated Product Evaluation of Chocolate Milk Samples

The samples were kept in the refrigerator and were served at about 4 °C in small white plastic cups [43] to panelists who were seated in individual sensory booths. In order to prevent bias, samples were given to the panelists one at a time in a random order. Sample containers were coded using three-digit random numbers. Panelists were asked to assess the samples using the prepared score sheet according to the descriptors created during the qualitative stage of the descriptive sensory analysis. Each attribute’s intensity was scored by the panelists by placing a vertical mark along the corresponding horizontal rating line. By measuring the distance from the line’s origin (“absent”) to the vertical mark, these markings were transformed into numerical data. In order to decrease the level of fatigue, panelists were given unsalted crackers and water in plastic cups. In order to check panelist performance, the evaluation was replicated four (4) times with afternoon and morning sessions for 2 days.

### 2.6. Consumer Evaluation of Chocolate Milk

The respondents in the chocolate milk evaluation were recruited mostly from the province of Nueva Ecija, Philippines, which is one of the provinces with the highest production of buffalo milk in the country. Eligibility for selection required that respondents should be regular consumers of chocolate or chocolate (based) drinks, have no specific disliking for chocolate drinks, have no food allergies or dietary intolerance, and be willing and available to participate in the study [44]. Since the consumer acceptability testing was conducted during the time of the COVID-19 pandemic, which prevented central location testing, respondents were visited individually in their homes. Standard consumer testing protocol was followed [42]. Respondents were oriented about the test and were asked to sign an Informed Consent form freely. Ethical approval was obtained prior to the conduct of the consumer testing. About 30 mL of chocolate milk was placed in a small plastic cup which was covered. The four test samples were identified and labelled using a randomly generated 3-digit code, and the sample temperature was about 4 °C. Two (2) enumerators interviewed the respondents in their respective houses to assess the four (4) samples of chocolate milk, presented one at a time using a randomized complete block design [45], based on their general acceptability and degree of liking on the sensory attributes of the product using a semi-structured questionnaire. A total of 120 respondents from the province of Nueva Ecija in the northern Philippines aged 21 to 68 years old evaluated the samples using a 9-point hedonic scale (where 1 = extremely dislike, 9 = extremely like) for overall and attribute liking. The attributes evaluated include color, aroma, taste, mouth feel, sweetness, bitterness, consistency, cocoa flavor, milk flavor, and aftertaste. The respondents were also asked about their opinions on the attributes of the chocolate milk using the 5-point Just-About-Right (JAR) scale, which combines intensity and hedonic judgement to determine the optimum attribute intensity level [46]. Consumer testing frequently includes the JAR scale to determine whether a product’s qualities are rated as either excessively high, excessively low, or about right and the direction and level of the needed correction [47]. They were also asked for attributes that they generally like or dislike in the chocolate milk. Lastly, they were asked about their purchase or buying intention using a 5-point scale (where 1 = definitely would not buy, 5 = definitely would buy).

### 2.7. Statistical Analysis

For compositional analysis of natural and alkalized cocoa powder, data were analyzed using an independent *t*-test, while a one-way analysis of variance (ANOVA) was conducted on the data from the physico-chemical and proximate composition of chocolate milk. For the descriptive sensory test, a three-way analysis of variance (products, panelists, and replication) was performed to determine which attributes discriminated between products and assess panel performance [48]. Tukey’s HSD multiple comparison tests were used to determine where the differences occur (α = 0.05) [49]. For consumer test data, two-way (products and respondents) ANOVA was performed on overall and individual attribute liking of chocolate milk samples. When significant effects were observed, the Tukey HSD post hoc test was used. Principal component analysis (PCA) was conducted to summarize the relationships between the chocolate milk samples and sensory attributes [50]. In order to assess the relationship between the overall acceptability and the sensory characteristics, partial least squares regression (PLSR) was run on the mean overall liking scores and mean descriptive sensory scores [51]. Standardized regression coefficient figures were produced for overall acceptance and the sensory characteristics that show the “drivers of liking” or the sensory characteristics that correlate either positively or negatively with overall acceptance. All analyses were performed using XLSTAT version 2023.1.1 software package (Addinsoft, New York, NY, USA).

## 3. Results and Discussion

### 3.1. Proximate Composition of Natural and Alkalized Cocoa Powders

A compositional analysis of the cocoa powders used for the prototype samples in this study is presented in Table 2. The two types of cocoa powders are significantly different (*p* < 0.05) in all components. Alkalized cocoa powder was higher in terms of ash and moisture content, while natural cocoa powder was higher in fat content. Natural cocoa powder has a lower pH value than alkalized cocoa powder which supports previous studies [52]. Based on the pH of the alkalized cocoa powder, it can be considered as heavily treated since it has a pH greater than 7.61 [53]. The pH of natural cocoa powder used in this study is in the neutral pH range. However, most natural cocoa powders are slightly acidic with a pH of 5.3–5.8 [53]. The acidity of cocoa beans, which is due to acetic acid produced during fermentation, and other volatile components may be affected by geographical origin, variety of cocoa beans, processing conditions, and drying conditions [54]. Artificial drying methods for cocoa beans sometimes produce under or over-dried cocoa, thereby causing the shell to solidify, trapping volatile acidic compounds inside the cocoa bean, and resulting in a high-acidity and low-chocolate flavor cocoa [55]. As such, the natural cocoa powder used in this study may have been derived from cocoa beans that were optimally fermented and dried and thus avoiding high acidity [56].

The moisture content of the two samples was relatively low, with a mean value of 2.33% and 4.29%. Cocoa powder is considered safe at a moisture content of up to 5% [57]. Total ash was higher in an alkalized cocoa powder (14.8%), which may have been affected by the type and quantity of compounds used in the alkalization process [28]. The ash content of non-alkalized cocoa powder is about 9% in another study [58]. Most commercially available cocoa powders have a fat percentage of between 10% and 24%. [59]. The crude fat value was relatively high in the natural cocoa powder sample, with a mean value of 19.63%, but it is still within the CODEX standard of 10–20% [60].

### 3.2. Proximate Content of Chocolate Milk Samples

The results obtained from the physico-chemical analysis of the chocolate milk are shown in Table 3. No significant differences were found for fat content and protein content (*p* > 0.05) among the chocolate milk samples. The fat contents of the chocolate milk samples ranged from 1.88% to 2.09%, while protein closely ranged from 2.48 to 2.84%. These values are comparable to those reported by [61], who investigated the chemical components of five commercial chocolate milk beverages. The differences in the total solids, solid non-fat, and lactose content for chocolate milk using alkalized and non-alkalized cocoa powders were statistically insignificant (*p* > 0.05) in the prototypes. However, there are noticeable discrepancies between the prototype and the commercial chocolate milk samples, which could be the result of different processing methods. The proximate content of the buffalo’s milk used for the prototype are within the range of those reported in the literature and are attached as Supplementary Material (Table S2. Comparison of proximate composition of raw buffalo milk used in the study with evidence from literature).

Color is one of the most significant aspects of quality that determines the acceptance or rejection of a product such as chocolate milk. Prototype alkalized chocolate milk (PA) had a hue of 5 YR, a value of 3, and a chromaticity of 4, revealing a darker brown chocolate milk which is expected since darker cocoa products are obtained when the pH is higher because an alkaline pH allows the polyphenol oxidase to accelerate the oxidation and polymerization of polyphenols, which results in a darker color [52]. In contrast, the prototype with natural cocoa powder had a hue of 7.5 YR, a value of 5, and a chromaticity of 4 which gave the sample a lighter brown color. The same trend was found in the commercial chocolate milk samples. Consumer research has shown that color significantly affects how flavor is perceived [62].

### 3.3. Sensory Profile of Buffalo Chocolate Milk

The four chocolate milk samples were analyzed by the trained panel (n = 8), and a total of 15 attributes were generated in terms of appearance (color, viscosity, and sandiness or the presence of cocoa particles in chocolate milk), aroma (cocoa, milky, and vanilla), texture by mouthfeel (smoothness, viscosity, and creaminess), taste and flavor (chocolate, milky, sweetness, and vanilla) and aftertaste (chocolate and bitterness). Table S1 (attached as Supplementary Material) shows the generated attributes and the definition adopted by the trained panel as well as the references that were used to assess the samples. After further training, the trained panel deemed that vanilla aroma and flavor were absent in all samples and it was removed from the final questionnaire.

Table 4 shows the mean intensity scores of the different sensory attributes of chocolate milk. ANOVA revealed that the samples differed significantly (*p* < 0.05) in terms of brown color. PA was significantly more intense (mean score: 12.1) in brown color than in the three samples. The difference may be attributed to the type and level of cocoa powder used in the formulation since the color varies based on these factors. Miller et al. [53] reported that natural cocoa powder has a typical light to medium brown color. In contrast, the color of chocolate milk with alkalized cocoa was obviously darker in color because the cocoa powder used had undergone an alkalization process, which counteracts the normal cocoa acidity and raises the pH up to 8 [63]. This result is consistent with the physical color analysis performed on cocoa powder. Color influences the perception of flavor identity and intensity [64], and it is one of the first aspects of quality that the human senses perceive, and consumers use it to judge the quality of food products [44].

Conversely, chocolate milk with alkalized cocoa significantly differed in terms of viscosity among the chocolate milk samples, having the highest mean value of 4.9. The amount of stabilizer(s) (i.e., hydrocolloids) is also responsible for the viscosity attribute in chocolate milk [65]. Furthermore, the samples are significantly distinguishable in terms of sandiness. The dosage of the stabilizer is a vital factor in the sandiness of chocolate milk; a little too low dosage will quickly produce undesirable levels of sedimentation [66].

In terms of cocoa aroma, PN had the highest mean score (7.4), which was significantly different from CA (6.4), PA (3.0), and CN (2.3). Cocoa aroma is one of the most important factors that affect consumer acceptability of cocoa-based milk products [67]. On the other hand, CA obtained the highest level of milky aroma (2.7), which was significantly higher (*p* < 0.05) than CN (2.0). Alkalization enables the breakdown of sugar and the production of Maillard reaction products (α-dicarbonyl compounds), which may have a favorable effect on the flavor, color, and aroma of cocoa [28,58].

CN was perceived to have the highest mean smoothness score (10.4), which was significantly distinguishable from CA (9.3), PN (5.1), and PA (5.7). The prototype chocolate milks were not significantly different (*p* < 0.05) from each other in terms of smoothness, having a mean value of 5.7 and 5.1, respectively. This was unexpected since the alkalized cocoa powder is more soluble compared to natural cocoa powder because alkali compounds can destroy ester linkages and subsequently hydrolyze cell walls in the cocoa powder matrices rendering it to be more soluble [68]. However, commercial chocolate milks had better smoothness than the two prototypes. The difference may be attributed to the machine (homogenizer) used during the processing of the two commercial samples. Consequently, PA was perceived by the panelists to have the highest intensity of viscosity (5.4), which was significantly different (*p* < 0.05) from the commercial chocolate milks and the prototype natural chocolate milk. CN obtained the highest mean score of creaminess (5.6), which was significantly distinguishable (*p* < 0.05) from the other samples. Consumer acceptability of chocolate milk depends on the product’s mouthfeel and texture, where certain dairy systems’ chalkiness, which is regarded as a mouthfeel flaw, may be modified by factors during processing, stabilizers, and ingredient quality [69].

In terms of flavor, PA had the highest intensity of chocolate flavor (8.9), which was significantly different from PN (8.1), and the commercial samples. CA obtained the highest milky taste (2.6), although it was not significantly different from CN (2.5) while significantly different (*p* < 0.05) from PN (2.0) and PA (1.6), respectively. PA obtained the highest mean sweetness intensity score (9.9), which was significantly different from commercial with alkalized CP (9.3), and the two chocolate milks with natural CP (8.9 and 9.2). Lowering the acidity makes the cocoa powder more easily incorporated into fats and liquids. Natural cocoa contains naturally occurring acids such as lactic, acetic, citric and oxalic [70].

Following the trend of flavor, PA obtained the highest mean intensity of chocolate aftertaste (8.0) which was not significantly different (*p* < 0.05) from PN (7.8) and CA (7.8) while it was significantly different from CN (3.9). Lastly, PA obtained the highest mean bitter aftertaste score (2.9), which was significantly higher than the other samples. Due to differences in salivary flow rates, various people have distinct perceptions of astringency and bitterness. As a result, preferences and acceptance of a product might vary greatly among people [71].

The descriptive sensory study confirmed previous research’s results that the sensory qualities of chocolate milk might vary depending on the type of cocoa used [35,72]. Alkalization is accomplished technologically by combining cocoa material with an alkali solution and then subjecting the resulting mixture to a combination of pressure and temperature [28]. The darker color of cocoa powder is one of the most obvious changes caused by alkalization. This is due to the Maillard reaction products, the oxidation and polymerization of polyphenols, their interactions with other molecules, and to a certain extent, polyphenol oxidase activity, which works better in basic conditions [35,73]. In terms of flavor, both volatile and non-volatile components contribute to cocoa flavor [54], and these components are significantly decreased in their absolute value by alkalization [28], which is supported by this study. Furthermore, solubility is one of the primary issues when adding cocoa powder to the recipes of various food products, such as milk beverages [28]. For powder solubility to increase, proteins, polyphenols, and their complexes must be released and destroyed, and this is remedied by alkalization [5]. However, since some polyphenols are destroyed, and their amount is significantly decreased [52], the nutritional value of chocolate milk is also affected.

### 3.4. Consumer Testing of Buffalo Chocolate Milk

Table 5 shows the general or overall acceptability scores of the different samples. Based on the results of the ANOVA, no significant difference was observed in the overall acceptability with respect to the types of cocoa powder. While all samples were equally liked, the incorporation of the cocoa powder in the buffalo milk may have masked the flavor differences in the cocoa powder.

#### 3.4.1. Hedonic Ratings of Individual Sensory Attributes

The mean liking of the specific sensory attributes of the chocolate milk samples is shown in Table 6. Statistically significant differences were observed for color, viscosity (mouthfeel), and chocolate flavor. Conversely, samples were statistically indistinguishable in terms of their mean liking ratings for aroma, visual viscosity, visual sandiness, smoothness, mouthfeel, and bitterness.

The result showed that in terms of appearance, PN has the lowest rating among the four when it comes to color, viscosity, and smoothness, while CA obtained the lowest rating in terms of viscosity. CN obtained the highest acceptability for color (8.0), which is statistically tied with the commercial alkalized (PCC) sample. Depending on the operating circumstances, alkalization can change the normal, natural cocoa color by combining the effects of alkali agents, water content, aeration, temperature, duration, and pressure to produce a cocoa color that ranges from light to dark and has red or black undertones [28].

When it comes to visual viscosity, the prototype with natural CP and the commercial with alkalized CP had the lowest rating among the four samples. However, there was no difference in liking the appearance in terms of viscosity between the types of cocoa powder. Viscosity in chocolate milk is mainly influenced by stabilizers such as alginate, xanthan gum, and carrageenan [74]. Only one type of stabilizer was used in the production of chocolate milk in this study which is carrageenan. In alkalized cocoa liquor, where cocoa power is derived, alkalization could also contribute to the increase in the viscosity of the resulting product [75].

The prototype with natural CP scored the lowest in visual sandiness, which indicates that there may be non-dissolved particles. Solubility is one of the key issues with using cocoa powder in the formulation of various food items, such as milk beverages [5]. In dairy, milk drinks use different types of hydrocolloids to stabilize the liquid system, and these affect the rheological properties of some beverages [54]. According to one study, a cocoa beverage made with alkalized cocoa powder is more stable than a beverage made with natural cocoa powder [74]. Due to the interconnected cell structures of cocoa proteins and polyphenols, they are thicker and more resistant to oxidation. Destroying these complexes and releasing these chemicals are, therefore, crucial goals in boosting powder solubility [5]. Alkalization may be one of the main factors that can increase the solubility of cocoa powder since it breaks ester links and hydrolyzes cell walls, and reduces fat content [68]. On the other hand, there is no significant difference in the appearance in terms of sandiness in the chocolate milk with the types of cocoa powder, whether alkalized or natural. Hydrocolloids are typically used to improve the stability, increase the creaminess, and lessen the graininess of chocolate milk [76]. However, excessive hydrocolloid usage can lead to unwanted characteristics, including coagulation, flocculation, and even sediment formation, because of the hydrocolloids’ strong interaction with milk proteins [77]. In terms of milky and cocoa aromas, the four samples are not significantly different. Cocoa flavor belongs to the volatile or fragrance group, which is sensed predominantly by nose receptors rather than oral taste buds. The complex biochemical and chemical processes that take place during the postharvest processing of raw beans give cocoa its distinctive aroma. These processes are influenced by a number of variables, such as the cocoa genotype, the chemical makeup of raw seeds, the environment, agricultural practices, processing, and manufacturing stages [78]. A study of alkalized cocoa powder found that it contained 2.69% of tetramethylpyrazine (chocolate), 3.22% isobutyl benzoate (green/fruity), and 1.38% linalool (floral) [58].

Mouthfeel is a very important aspect of the quality of many food products, and it is thus associated with consumer acceptability [79]. For smoothness and creaminess, there are no significant different types of cocoa powder. The term “creaminess” refers to the milk fat globules that give dairy products their characteristic thickness, smoothness, and mouth-coating taste [80,81]. There is a significant difference in mouthfeel in terms of viscosity. The two buffalo chocolate milk samples with alkalized cocoa powder were statistically different from each other in terms of viscosity liking. This may be partially due to differences in formulation since one is commercial and the other is a prototype.

In terms of chocolate aftertaste with the types of cocoa powder, a significant difference was found, while the bitter aftertaste is not distinguishable among products. Caffeine, theobromine, methylxanthines, diketopiperazines, monomeric and oligomeric flavan-3-ols, and diketopiperazines (formed by the thermal breakdown of proteins during cocoa roasting and alkalization) are all associated with cocoa bitterness [82].

#### 3.4.2. Just-About-Right Ratings

In this study, all attributes of the samples, regardless of alkalization of cocoa powder, were judged by the consumer respondents to be at their optimum or just-about-right level (70-85%), namely: color, viscosity, mouthfeel, sweetness, chocolate flavor, and bitter aftertaste. Figures S1–S11 (attached as Supplementary Material) shows the Just about Right scores of buffalo chocolate milk samples evaluated by consumers. A minimum of 70% of responses in the JAR assessment are usually required to ascertain that an attribute is optimally aligned, while a minimum of 20% of consumers in the “too weak” or “too strong” categories is usually required to establish that an attribute is not at its optimal level [83]. Figures S12–S15 (attached as Supplementary Material) shows the mean drop plots of the samples.

### 3.5. Relationship between Sensory Descriptors and Consumer Acceptability

**Figure 1:**
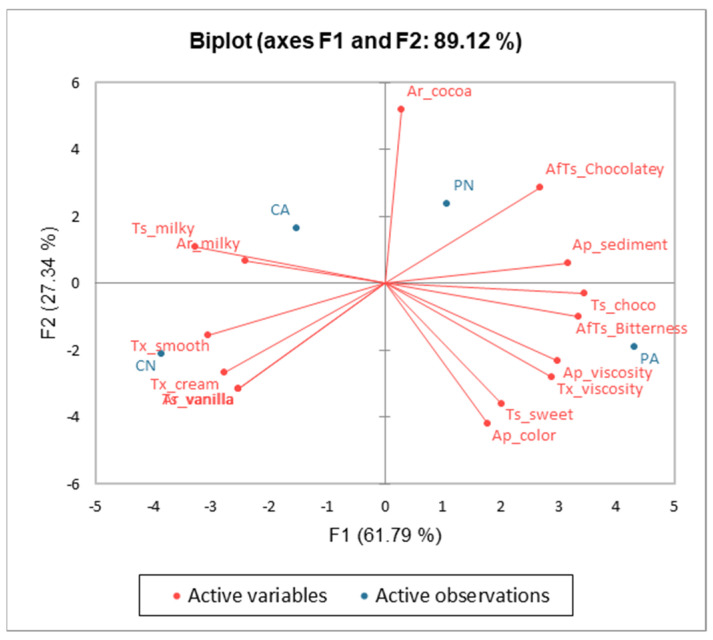
PCA loadings and scores of the sensory attributes and the four buffalo chocolate milk samples.

Principal Component Analysis (PCA) was conducted using the trained panelist’s data [84]. PCA showed that the two principal components, 1 (F1) and 2 (F2), explained 61.79% and 27.34% of the overall variance (89.12%), respectively (Figure 1). The first dimension consists mainly of chocolate flavor, bitter aftertaste, viscous appearance, and chocolate aftertaste, which are associated with cocoa powder. The second factor was defined by attributes such as milky aroma and milky taste, smoothness, and creaminess which are inherent characteristics of milk.

**Figure 2:**
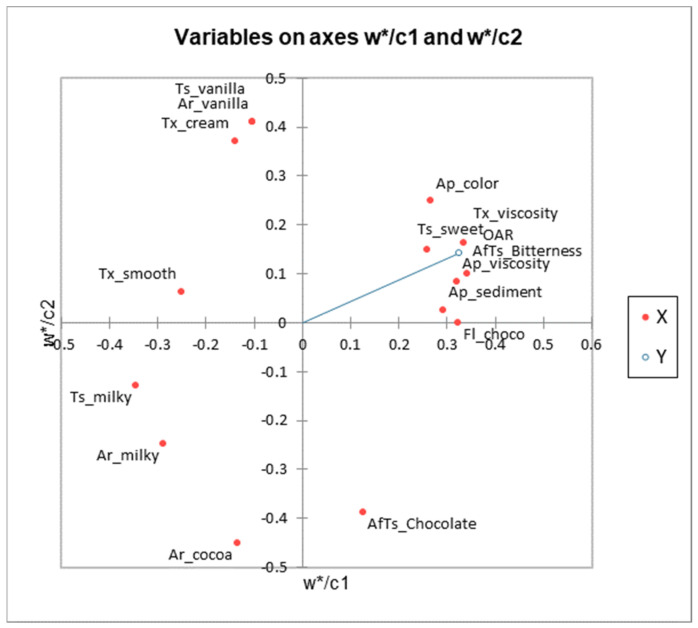
PLSR chart showing the relationship between the sensory characteristics of the chocolate milk samples and consumer acceptance.

Partial least-squares regression (PLSR) was used to determine the relationship between descriptive sensory data and consumer acceptance data (Figure 2) [50,85]. The X data set (explanatory variables) for PLSR consisted of the mean ratings for the descriptive ratings, and the Y data set (consumer acceptability ratings) consisted of the mean ratings for overall acceptability) [50]. With the use of PLSR, characteristics that are favorably or adversely connected with general acceptability may be found [83].

**Figure 3:**
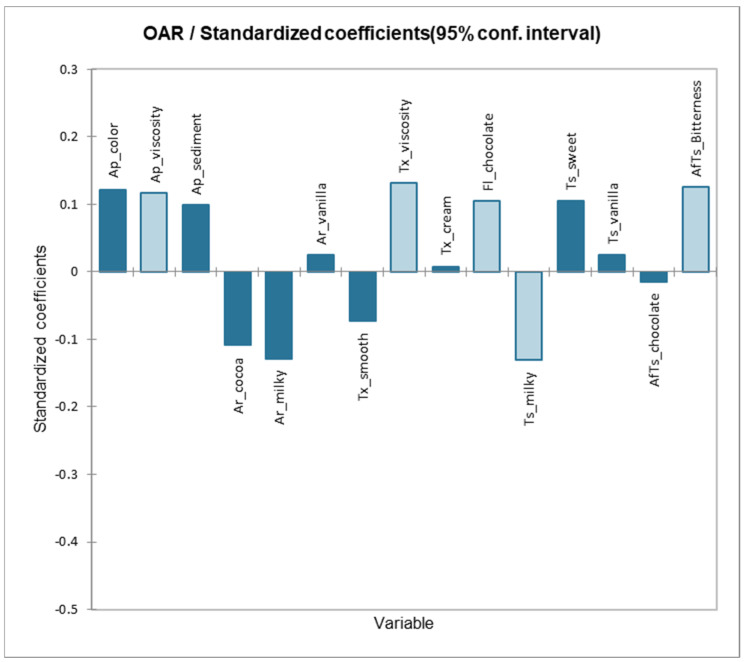
Standardized regression coefficients for sensory attributes predicting overall liking of chocolate milk based on partial least square regression. Light blue bars indicate significant effect (α = 0.05) of this sensory attribute on the overall liking score.

PLSR provided a binary factor model based on a statistic *Q*^2^, which measures the importance of a PLS component for predicting the whole set of Y [86]. Due to the small number of products compared to other fields, two components in the sensory analysis are typically adequate [85]. Standardized regression coefficients (Figure 3) show that significant positive “drivers of liking” (indicated by light blue bars) are viscous appearance (Ap_viscosity), viscous mouthfeel (Tx_viscosity), chocolatey flavor (Fl_chocolate), and bitter aftertaste (AfTs_Bitterness). Some of these attributes were also identified as drivers of liking in a previous study on bovine chocolate milk [67]. If a product performs well on these criteria, total liking will rise, and these liking drivers will alter overall liking when the strength of the physical stimulus increases [83].

Furthermore, the only significant negative driver of liking is the milky taste. This may be due to the inherent content of odor-active volatile compounds detected in a previous study in buffalo milk, such as octen-3-ol (mushroom), nonanal (grassy), indole (stable animals), and an unidentified compound characterized by a smoked cheese aroma [87]. A cursory inspection of the goodness of fit statistics of the PLS model shows that cumulative *Q^2^* = 0.80, indicating a high degree of stability in the model, and the difference between R^2^ and *Q^2^* was below 0.3, indicating that the PLSR model had a high explanatory value [51,88].

### 3.6. Purchase Intention

Table 7 shows the purchase intention for buffalo chocolate milk with different types of cocoa powder. All four samples scored high (mean > 4) when it comes to consumers’ purchase intention. High scores denote the high marketability of the sample, while low scores denote poor marketability. ANOVA revealed that there was no significant difference among the samples, which confirms that all samples were equally liked.

### 3.7. Implications and Limitations

The research findings revealed that natural and alkalized cocoa powder showed similar performance in terms of the acceptability of buffalo chocolate milk. As the production of natural cocoa powder is relatively less expensive, smallholder cocoa farmers and microenterprises producing cocoa powder may benefit from these results. Thus, natural cocoa powder can be an affordable and more available flavor additive to water buffalo milk, especially in developing countries. Although the descriptive analysis showed sensory differences for 8 out of 13 attributes, consumers were only able to differentiate the samples based on their liking of three attributes, namely: color, viscosity, and chocolate flavor. This may be due to the more integrated way of evaluation by consumers in an affective test compared to the more analytical mindset of the trained panel. Customers often evaluate their liking or disliking of a product as a whole based on the integrated pattern of sensory input they receive from it [42]. While prototype chocolate milk with alkalized cocoa powder was significantly more intense in these three attributes, the differences may not be strong enough to stand out based on the hedonic ratings. The intensity of those attributes was expected because alkalization causes the cocoa powder to become darker [89] and form more viscous products [41,75]. The only attribute where the magnitude of the difference was more apparent was color. However, based on PLSR, intense color provided by alkalization was not a significant driver of liking, which supports the finding of a previous study that dark color does not necessarily drive consumer liking of chocolate milk [67]. As such, the more intense brown color produced by alkalization [41] is not pertinent in buffalo chocolate milk. Moreover, since consumers generally prefer and are willing to pay more for organic or natural products [37,39], the use of natural cocoa powder in dairy beverages can be a marketing advantage, especially for buffalo chocolate milk. Moreover, using natural cocoa powder may be more beneficial in terms of nutrition since alkalized cocoa powder has lower phytochemical contents [29,30]. Furthermore, since the milky taste was identified as a significant negative driver of liking, which may be due to the inherent gamy flavor in buffalo milk [87], increasing the amount of cocoa powder could be a solution to mask the typical buffalo milk flavor but should be kept at a level where sedimentation will not easily occur [5].

While careful steps were taken in order to control possible bias, such as ensuring the uniformity of formulation in the chocolate milk samples and thorough training of the descriptive panel [42,44], this study has limitations. This study was confined to cold-consumed chocolate milk from buffalo milk that is available in the Philippine market. This is different from hot chocolate, which is often produced by combining pure cocoa powder, a cocoa powder and sugar blend, chocolate flakes, or block chocolate in either milk or water [23]. Cold-consumed chocolate milk was selected because ready-to-drink refrigerated chocolate milk is the most popular among children and adults [67,88]. However, future studies could also investigate the effect of alkalized and natural cocoa powder on hot chocolate beverages. While statistical power was ensured by employing a high number of respondents, future studies may include a broader number of prototypes while investigating the impacts of varying levels of cocoa powder. Although a stabilizer was included in the formulation of buffalo chocolate milk [5], a sedimentation test was not included in this study. Lastly, the addition of other complementary flavors to chocolate milk, such as cinnamon which contains bioactive compounds [89], may increase the nutritional quality of the product. A consumer study on the effect of nutrition information (i.e., use of natural cocoa powder) and how it could increase liking and preference of chocolate milk beverages may be conducted in future studies.

## 4. Conclusions

Based on the results, this study provided rich and extensive data on the effects of alkalized and natural cocoa powders on the physico-chemical, sensorial, and consumer acceptability of buffalo chocolate milk. Descriptive analysis showed that 8 out of 13 attributes are good discriminators of product differences. Although the overall acceptability of all samples was high and statistically tied, hedonic ratings of the individual attributes revealed that only consumer liking for color, viscosity (mouthfeel), and the chocolate flavor differed between samples. Combining consumer acceptability data and sensory descriptive data, positive “drivers of liking” were identified: chocolate flavor, viscous appearance, viscous mouthfeel, and bitter aftertaste. The darker color provided by alkalized cocoa powder did not drive consumer liking. Lastly, all four samples scored equally high on purchase intention. Buffalo chocolate milk with natural and alkalized cocoa powder, both in commercial and prototype forms, were liked equally by consumers. Since natural cocoa powder is less costly to produce, it will be beneficial to smallholder farmers and microenterprises that are producers of cocoa powder and cocoa farmers in developing countries.

## Figures and Tables

**Table 1:** Chocolate milk samples used in the sensory evaluation and consumer testing.

Sample Code	Cocoa Powder (CP) Type	Brand/Cocoa Powder
CA	Commercial chocolate milk with alkalized CP	PCC, Science City of Munoz, Philippines
CN	Commercial chocolate milk with natural CP	DVF, Nueva Ecija, Philippines
PA	Prototype chocolate milk with alkalized CP	Valmarce cocoa powder, Bulacan, Philippines
PN	Prototype chocolate milk with natural CP	Ricoa cocoa powder, Pasig City, Philippines

**Table 2:** Characteristics of cocoa powders.

Component	Alkalized Cocoa Powder	Natural Cocoa Powder
Ash (%)	14.79 ^a^ ± 0.36	7.99 ^b^ ± 0.06
Fat (%)	10.90 ^b^ ± 0.00	19.63 ^a^ ± 0.15
Moisture content (%)	4.29 ^a^ ± 0.01	2.33 ^b^ ± 0.11
pH	8.37 ^a^ ± 0.02	7.02 ^b^ ± 0.01

Note: Means having different superscripts within the row are significantly different at *p* < 0.05; Values are means ± standard deviation of three samples.

**Table 3:** Proximate analysis of water buffalo chocolate milk with alkalized and natural cocoa powder.

Sample Code	PA	PN	CN	CA
Fat	1.88 ^a^	2.09 ^a^	1.51 ^a^	1.72 ^a^
Lactose	7.57 ^b^	7.76 ^b^	8.93 ^a^	7.19 ^c^
Protein	2.73 ^a^	2.84 ^a^	2.48 ^a^	2.57 ^a^
Total Solids	15.64 ^b^	16.10 ^b^	16.78 ^a^	14.65 ^c^
Solid Non-Fat	13.67 ^b^	14.01 ^b^	14.75 ^a^	13.00 ^c^

Note: Means having different superscripts within the same row are significantly different at *p* < 0.05; Values are means of three samples. PA—Prototype with alkalized CP; PN—Prototype with natural CP; CN—Commercial with natural CP; CA—Commercial with alkalized CP.

**Table 4:** Mean intensity scores of the chocolate milk samples.

Sample Code	PA	PN	CN	CA
*Appearance*				
Color	12.1 ^a^	2.2 ^d^	4.5 ^c^	6.4 ^b^
Viscosity	4.9 ^a^	3.4 ^b^	3.5 ^b^	3.3 ^b^
Sandiness	6.6 ^a^	6.4 ^a^	2.8 ^b^	2.6 ^b^
*Aroma*				
Cocoa Aroma	3.0 ^c^	7.5 ^a^	6.4 ^b^	2.4 ^d^
Milky Aroma	1.1 ^c^	1.1 ^c^	2.8 ^a^	2.0 ^b^
*Texture (Mouthfeel)*				
Smoothness	5.7 ^c^	5.1 ^c^	9.3 ^b^	10.5 ^a^
Viscosity	5.5 ^a^	4.0 ^b^	3.9 ^b^	4.0 ^b^
Creaminess	1.9 ^b^	2.2 ^b^	2.3 ^b^	5.7 ^a^
*Taste and flavor*				
Chocolate	8.9 ^a^	8.1 ^b^	7.3 ^c^	7.0 ^c^
Milky	1.6 ^c^	2.0 ^b^	2.6 ^a^	2.5 ^a^
Sweetness	9.9 ^a^	9.0 ^b^	9.3 ^b^	9.2 ^b^
*Aftertaste*				
Chocolate	8.0 ^a^	7.8 ^a^	7.7 ^a^	4.0 ^b^
Bitterness	2.9 ^a^	2.4 ^b^	1.8 ^c^	1.8 ^c^

Note: Means having different superscripts within the same row are significantly different at *p* < 0.05; Values are means scores of four replications. PA—Prototype with alkalized CP; PN—Prototype with natural CP; CN—Commercial with natural CP; CA—Commercial with alkalized CP.

**Table 5:** Mean overall acceptability scores of the chocolate milk samples.

Sample Code	PN	PA	CN	CA
Overall Acceptability (Mean ± SD)	7.89 ^a^ ± 1.56	8.02 ^a^ ± 1.10	8.27 ^a^ ± 0.99	7.97 ^a^ ± 1.28

Note: Means having different superscripts within the same row are significantly different at *p* < 0.05; Values are means ± standard deviation (n = 120). PA—Prototype with alkalized CP; PN—Prototype with natural CP; CN—Commercial with natural CP; CA—Commercial with alkalized CP.

**Table 6:** Mean liking scores of the sensory attributes of buffalo chocolate milk.

Sample	Appearance			Aroma		Mouthfeel			Taste and flavor	
	Color	Viscosity	Sandiness	Cocoa	Milky	Smoothness	Viscosity	Creaminess	Chocolate	Bitterness
PA	7.9 ^b^	7.6 ^a^	7.6 ^a^	7.7 ^a^	7.8 ^a^	7.8 ^a^	7.7 ^b^	7.8 ^a^	8.1 ^b^	7.3 ^a^
PN	7.6 ^a^	7.3 ^a^	7.4 ^a^	7.6 ^a^	7.7 ^a^	7.7 ^a^	7.3 ^ab^	7.6 ^a^	7.7 ^a^	6.8 ^a^
CN	8.0 ^ab^	7.8 ^a^	7.7 ^a^	8.0 ^a^	7.9 ^a^	8.0 ^a^	7.8 ^ab^	8.1 ^a^	8.3 ^ab^	7.5 ^a^
CA	7.5 ^ab^	7.4 ^a^	7.3 ^a^	7.7 ^a^	7.8 ^a^	7.7 ^a^	7.7 ^a^	8.0 ^a^	7.8 ^a^	7.2 ^a^

Note: Means having different superscripts within the same column are significantly different at *p* < 0.05; Values are mean scores (n = 120). PA—Prototype with alkalized CP; PN—Prototype with natural CP; CN—Commercial with natural CP; CA—Commercial with alkalized CP.

**Table 7:** Mean score on the purchase intention of respondents towards buffalo chocolate milk.

Sample Code	PN	PA	CN	CA
Purchase intention	4.4 ^a^ ± 0.7	4.6 ^a^ ± 0.8	4.5 ^a^ ± 0.6	4.4 ^a^ ± 0.9

Note: Means having different superscripts within the same row are significantly different at *p* < 0.05; Values are mean scores ± standard deviation (n = 120) with (1 = definitely will not buy; 5 = definitely will buy). PA—Prototype with alkalized CP; PN—Prototype with natural CP; CN—Commercial with natural CP; CA—Commercial with alkalized CP.

## Data Availability

The consumer testing data are not publicly available since participants did not provide their consent to share their data.

## Supplementary Materials

The following supporting information can be downloaded at: https://www.mdpi.com/article/10.3390/foods12091797/s1, Table S1: Definition of attributes and references in the evaluation of the chocolate milk samples; Table S2. Comparison of proximate composition of raw buffalo milk used in the study with evidence from literature [18,59,60]; Figures S1–S11. Just about right scores of buffalo chocolate milk samples evaluated by consumers; Figures S12 and S13. Mean drop plot of all the prototype chocolate milk products; Figures S14 and S15. Mean drop plot of all the commercial chocolate milk products; Figures S16. Variable Importance in the Projection.
