# Supplementary material for: Physico-Chemical Property, Sensory Profile and Consumer Acceptability of Water Buffalo (Bubalus bubalis L.) Chocolate Milk Using Alkalized and Natural Cocoa Powder
Source: Foods. 2023 Apr 26;12(9):1797. doi: 10.3390/foods12091797 (PMC10178308; doi:10.3390/foods12091797)
Supplement: Supplementary file 1 [file foods-12-01797-s001.zip › foods-2316369-supplementary.pdf]

Supplementary material

To accompany

Physico-chemical Property, Sensory Profile and Consumer Acceptability of Water Buffalo (*Bubalus bubalis* L.) Chocolate Milk using Alkalized and Natural Cocoa Powder

Joel G. Juvinal [1,2,3], Hans De Steur [1,2,*], Joachim J. Schouteten [1,2], Dimas Rahadian Aji Muhammad [4], Alma A. de Leon [3], Koen Dewettinck [2,5] and Xavier Gellynck [1,2]

[1] Department of Agricultural Economics, Ghent University, Coupure Links 653, 9000 Ghent, Belgium
[2] Sensolab, Faculty of Bioscience Engineering, Ghent University, Coupure Links 653, 9000 Ghent, Belgium
[3] Department of Food Science and Technology, College of Home Science and Industry, Science City of Munoz 3120, Nueva Ecija, Philippines
[4] Department of Food Science and Technology, Universitas Sebelas Maret (UNS), Jl. Ir Sutami 36A Kentingan Jebres, 57126 Surakarta, Indonesia
[5] Department of Food Technology, Safety and Health, Food Structure & Function Research Group (FSF), Faculty of Bioscience Engineering, Ghent University, Coupure Links 653, 9000 Ghent, Belgium
* Correspondence: hans.desteur@ugent.be (H.D.S.); Tel.: +32-92645945; , 9000 Ghent, Belgium

Table S1. Definition of attributes and references used by the trained panel to evaluate the chocolate milk samples

Attribute	Definition	Reference
Appearance		
1. Brown color	The intensity of brown color from light to dark brown	Munsel Color File<br>Light brown: 7.5 YR 6/2<br>Dark brown: 7.5 YR 4/2
2. Viscosity	Thick or sticky liquid or not flowing easily	Cooking oil (Minola)
3. Visual sediments	The amount of solid particles settling at the bottom	Midpoint: 1% cocoa powder solution (Bensdorp cocoa powder)<br>Intense: 3% cocoa powder solution (Bensdorp cocoa powder)
Aroma		
4. Cocoa aroma	The aromatic associated with cocoa powder	Midpoint: 1% cocoa powder solution (Bensdorp cocoa powder)<br>Intense: 3% cocoa powder solution (Bensdorp cocoa powder)
5. Milky aroma	The aromatic associated with fresh or pasteurized milk	Midpoint: 80% sterilized milk (Bear Brand, Nestle, Philippines)<br>Intense: 100% sterilized milk (Bear Brand, Nestle, Philippines)
Texture (Mouthfeel)		

6. Smoothness	Degree to which sample is free of grittiness	Lacking: Chuckie chocolate milk (Nestle, Philippines)<br>Intense: 3% cocoa solution
7. Viscosity	Thick or sticky liquid not flowing easily	Midpoint: Chuckie chocolate milk (Nestle, Philippines)
8. Creaminess	The mouth-feel related to the smoothness of the chocolate as related to fat	Intense: Nestle cream (Nestle, Philippines)
Flavor		
9. Chocolate	The taste on the tongue associated with chocolate	Intense: Dark chocolate (Hershey's)
10. Milky	The taste on the tongue associated with vanilla extract	Midpoint: 80% sterilized milk (Bear Brand, Nestle, Philippines)<br>Intense: 100% sterilized milk (Bear Brand, Nestle, Philippines)
11. Sweetness	The taste on the tongue associated with table sugar	Midpoint anchor: 5% sucrose solution (Victoria pure refined sugar, Philippines)<br>Intense: 10% sucrose solution (Victoria pure refined sugar, Philippines)
Aftertaste		
12. Bitterness	The residual taste on the tongue associated with caffeine	Intense: Dark chocolate (Hershey's)
13. Chocolate	The residual taste on the tongue associated with chocolate	Intense: Dark chocolate (Hershey's)

Table S2. Comparison of proximate composition of raw buffalo milk used in the study with evidence from literature

Component	Mean content (in %)	Published values (in %) (reference)
Fat	13.68 ± 0	5.3 – 9 [59]
Lactose	4.44 ± 0	3.2 – 4.9 [59]
Protein	3.19 ± 0.02	2.7 – 4.6 [59]
Total solids	22.25 ± 0.02	19.03 [60]
Solid non-fat	8.57 ± 0.02	9.48 [18]

18. Kapadiya, D.B.; Prajapati, D.B.; Jain, A.K.; Mehta, B.M.; Darji, V.B.; Aparnathi, K.D. Comparison of Surti Goat Milk with Cow and Buffalo Milk for Gross Composition, Nitrogen Distribution, and Selected Minerals Content. *Vet. World* **2016**, *9*, 710–716.https://doi.org/10.14202/vetworld.2016.710-716.
59. Della Lucia, F.; Do Carmo, J.R.; Morais, C.S.N.; Nunes, C.A.; Pinheiro, A.C.M.; Ferreira, E.B.; Pinto, S.M.; De Abreu, L.R.; Vilas Boas, E.V. de B. Physicochemical and Sensory Quality of Several Commercial Brazilian Chocolate Milk Beverages. *Int. J. Dairy Technol.* **2016**, *69*, 364–371.
60. Shankar, M.U.; Levitan, C.A.; Prescott, J.; Spence, C. The Influence of Color and Label Information on Flavor Perception. *Chemosens. Percept.* **2009**, *2*, 53–58.

Appearance attributes

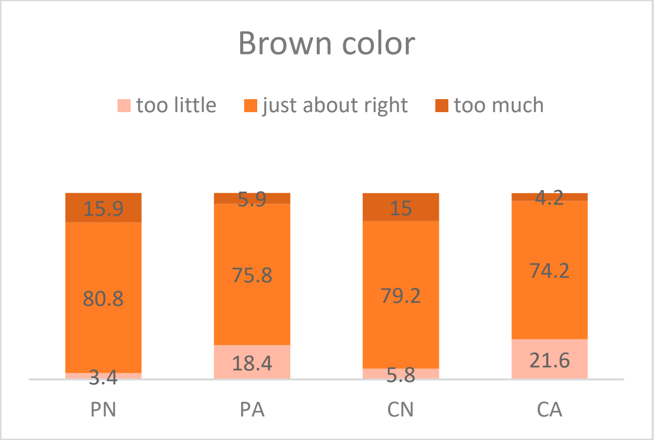

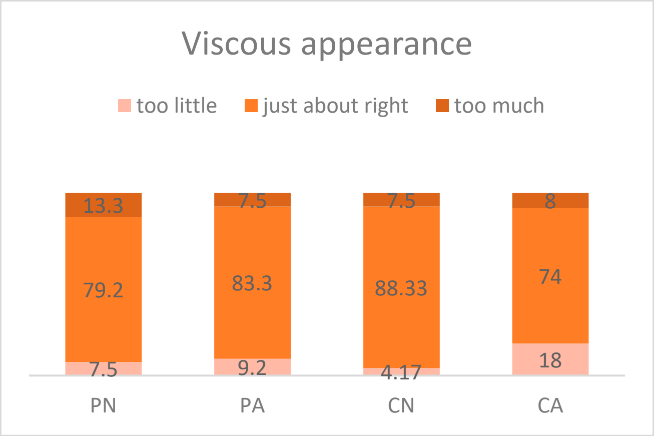

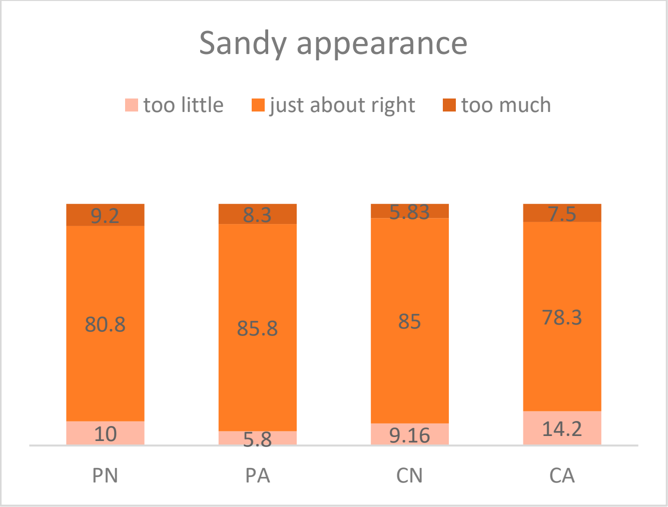

Aroma attributes

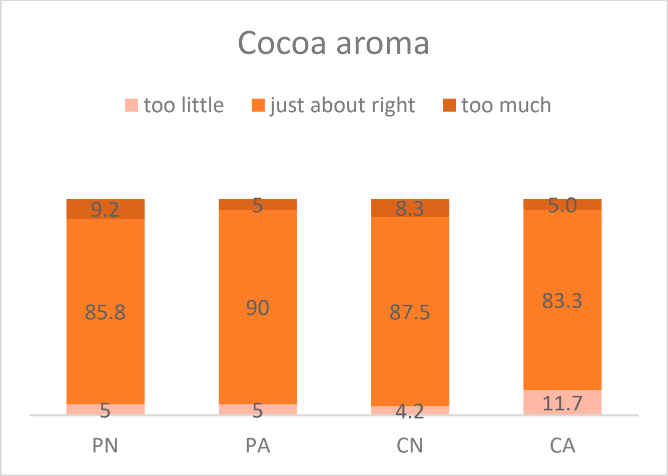

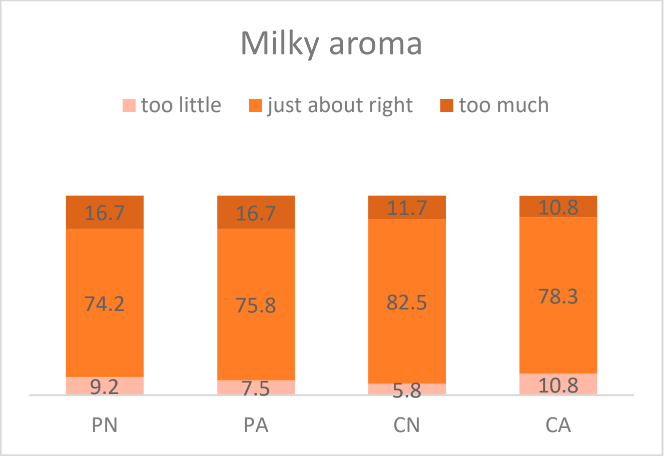

Texture attributes

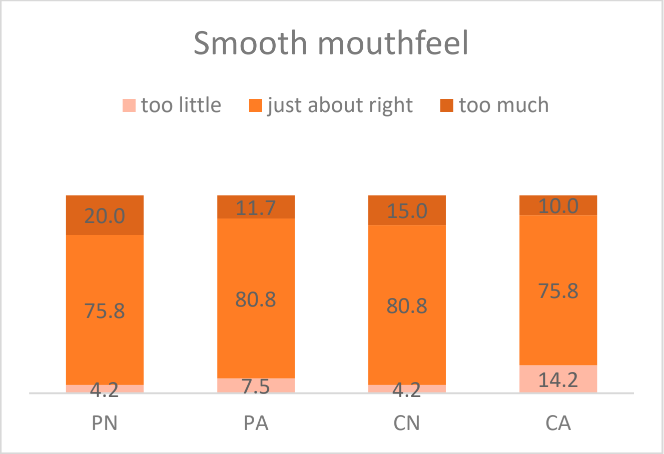

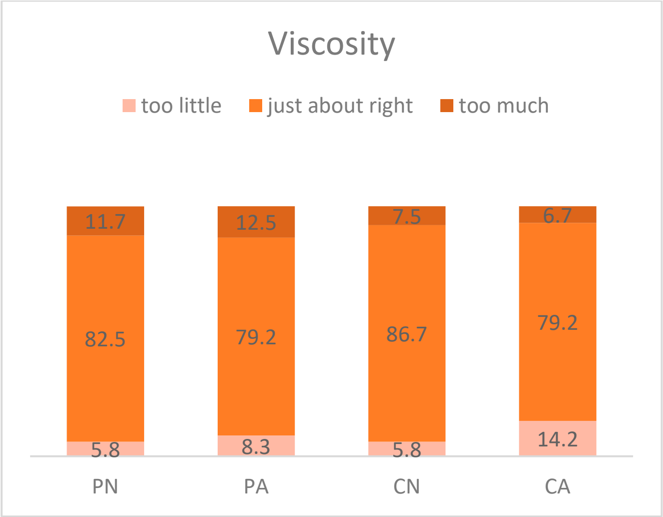

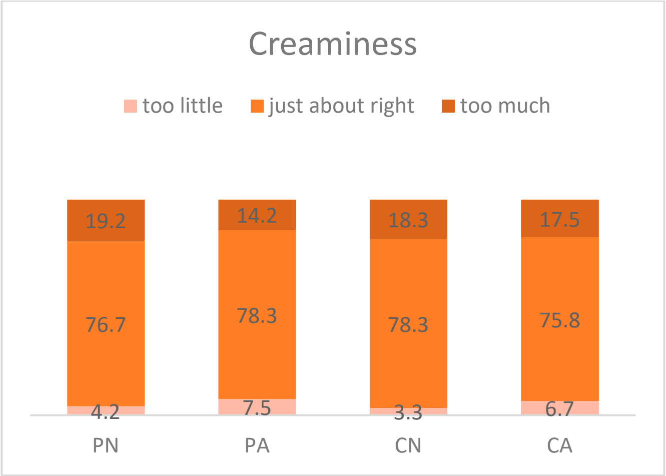

Taste and aftertaste attributes

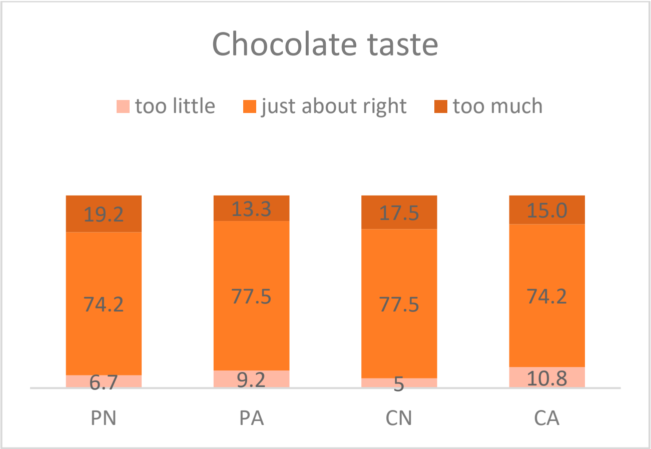

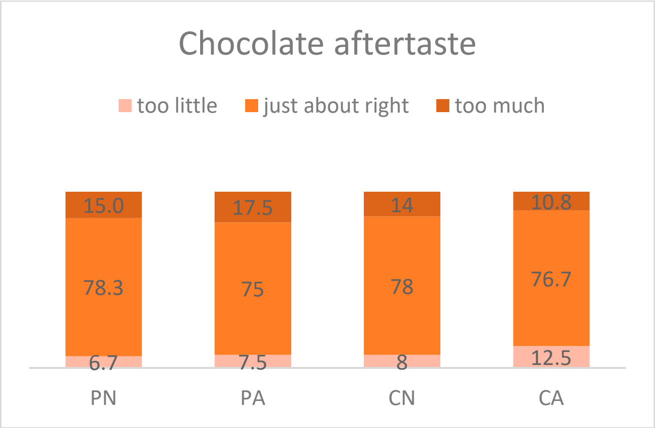

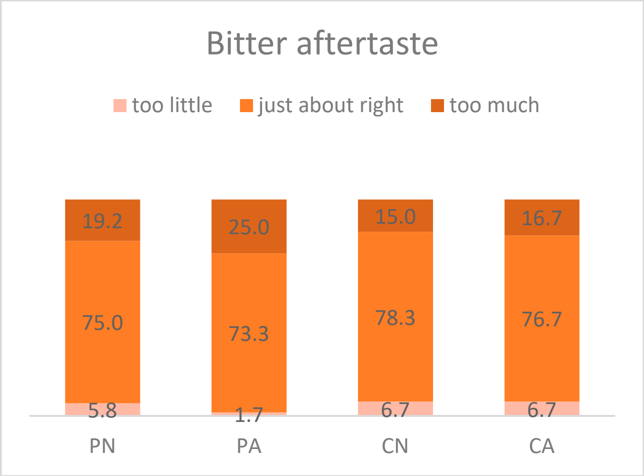

Figures S1–S11. Just about right scores of buffalo chocolate milk samples evaluated by consumers (n = 120). Values are percentages.
Note: PA - Prototype with alkalized CP; PN - Prototype with natural CP; CN - Commercial with natural CP; CA - Commercial with alkalized CP

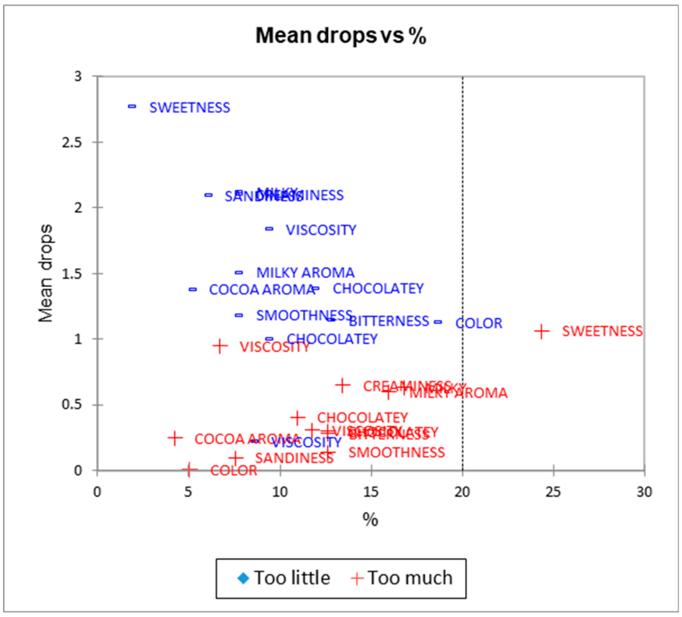

PN

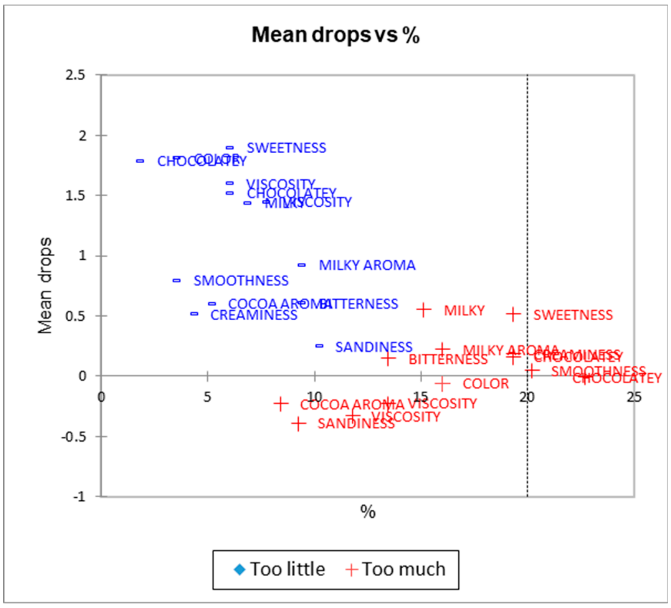

PA

Figures S12-S13. Mean drop plot of all the prototype chocolate milk products.
The 'too low' endpoint of the JAR scales are highlights with red, the 'too strong' endpoint is highlighted with blue. The dashed line represents the 20 % of the consumers.

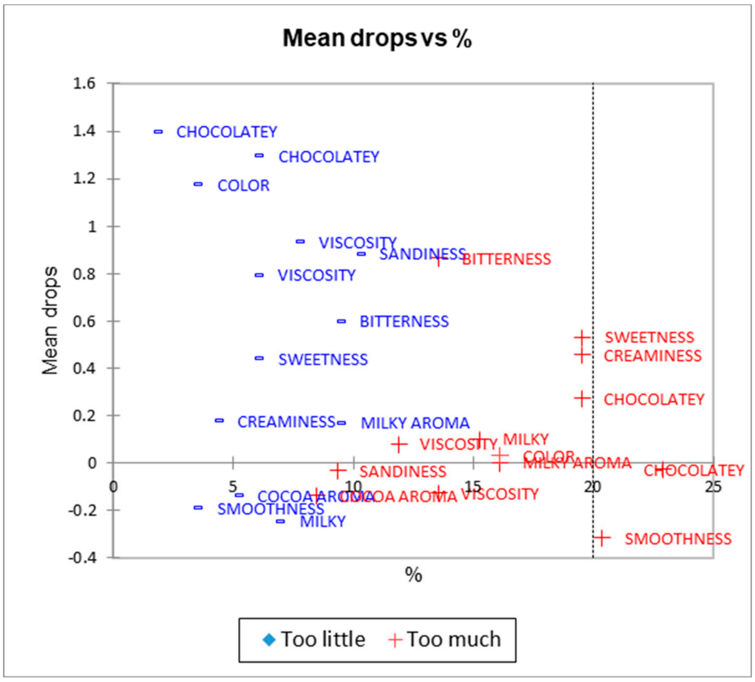
Mean drops vs %
Mean drops
%
1.6
1.4
1.2
1
0.8
0.6
0.4
0.2
0
-0.2
-0.4
0
5
10
15
20
25
CHOCOLATEY
CHOCOLATEY
COLOR
VISCOSITY
SANDINESS
BITTERNESS
VISCOSITY
BITTERNESS
SWEETNESS
CREAMINESS
SWEETNESS
CREAMINESS
CHOCOLATEY
MILKY AROMA
VISCOSITY
MILKY
SANDINESS
CHOCOLATEY
COCOA AROMA
COCOA AROMA
VISCOSITY
SMOOTHNESS
MILKY
SMOOTHNESS
Too little
Too much
CA

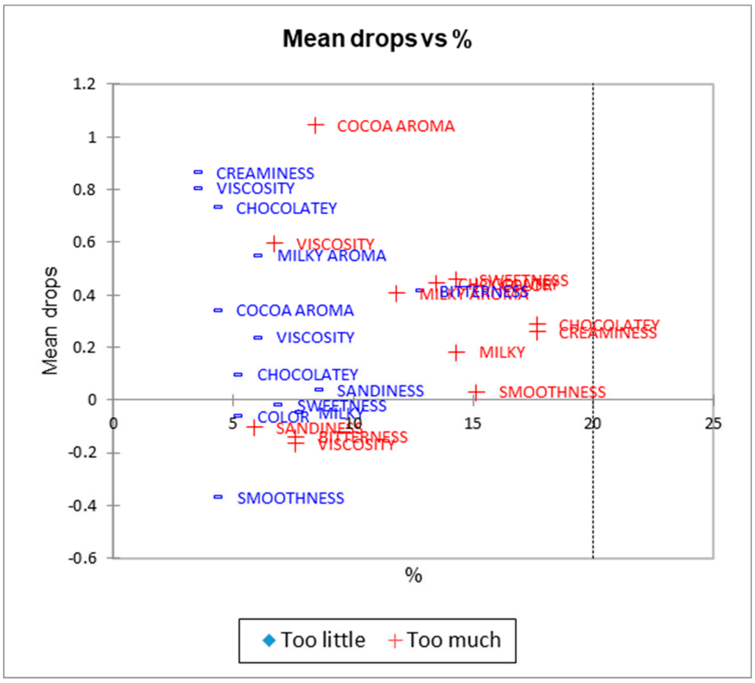
Mean drops vs %
Mean drops
%
1.2
1
0.8
0.6
0.4
0.2
0
-0.2
-0.4
-0.6
0
5
10
15
20
25
COCOA AROMA
CREAMINESS
VISCOSITY
CHOCOLATEY
VISCOSITY
MILKY AROMA
COCOA AROMA
VISCOSITY
CHOCOLATEY
CREAMINESS
MILKY
CHOCOLATEY
SANDINESS
SMOOTHNESS
SWEETNESS
COLOR
MILKY
SANDINESS
BITTERNESS
VISCOSITY
SMOOTHNESS
Too little
Too much
CN

Figures S14-S15. Mean drop plot of all the commercial chocolate milk products.
The 'too low' endpoint of the JAR scales are highlights with red, the 'too strong' endpoint is highlighted with blue. The dashed line represents the 20 % of the consumers.

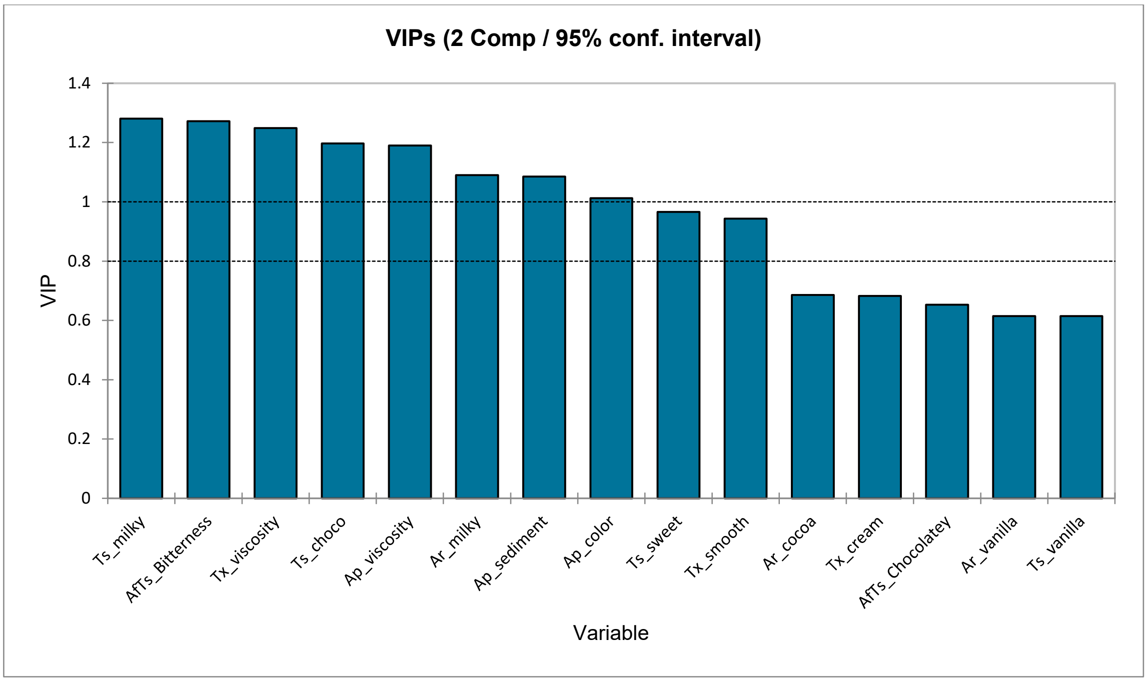

Figures S16. Variable Importance in the Projection (n=120)
